# Efficient N_2_- and O_2_-Sensing Properties of PtSe_2_ With Proper Intrinsic Defects

**DOI:** 10.3389/fchem.2021.676438

**Published:** 2021-05-24

**Authors:** Xin Yong, Jianqi Zhang, Xiangchao Ma, Weiming He

**Affiliations:** School of Physics and Optoelectronic Engineering, Xidian University, Xi’an, China

**Keywords:** gas sensing, electronic structures, optical properties, first-principles calculations, intrinsic defects

## Abstract

Developing efficient N_2_ and O_2_ gas sensors is of great importance to our daily life and industrial technology. In this work, first-principles calculations are performed to study the N_2_ and O_2_ gas-sensing properties of pure and defected PtSe_2_. It is found that both N_2_ and O_2_ adsorb weakly on pure PtSe_2_, and adsorption of the molecules induces negligible changes in the electrical and optical properties. Whereas the Pt@Se anti-site defect significantly improves the N_2_ adsorption capacity of PtSe_2_ and induces notable changes in the electrical property. Similar results are also observed for the Pt and Se vacancies and Pt@Se anti-site defects when examining O_2_ adsorption. In addition, notable changes in the optical absorption spectra of the PtSe_2_ with Pt@Se defect are induced upon N_2_ adsorption, which also occurs for PtSe_2_ with Pt and Se vacancies and Pt@Se anti-site defects upon O_2_ adsorption. These results demonstrate that PtSe_2_ with the corresponding defects can be both excellent electrical and optical sensors for detecting N_2_ and O_2_ gases. Our work offers a new avenue for preparing efficient gas sensors.

## Introduction

Oxygen is not only essential to the lives of humans and animals but also the key to the combustion-dependent processes such as power generation, chemical compound production, and heating. Controlling the air-to-fuel ratio during the combustion process at the critical point of excess oxygen is beneficial for improving the combustion efficiency, product generation, and safe combustion (Shuk and Jantz, 2015; Zhang et al., 2017). Because the presence of O_2_ corrodes gas storage and transportation systems, monitoring O_2_ in biomethane is also a necessary part (Urriza-Arsuaga et al., 2019). In the medical and food processing and waste management industries, sometimes it is also necessary to measure the oxygen content (Hong et al., 2018; Wang et al., 2019). Therefore, an efficient sensor for detecting O_2_ molecules plays an important role in modern technology. On the other hand, biogas is believed to be a promising substitute for natural gas due to its high methane content. However, the presence of impurity gas like N_2_ leads to a lower heating value. In addition, fuel dilution with N_2_ gas is generally used to reduce heat radiation, which is one of the main factors limiting the efficiency of gas turbines and internal combustion engines. Therefore, in order to meet the quality specifications, it is also necessary to detect and control N_2_ gas (Yi et al., 2013).

**GRAPHICAL ABSTRACT:**
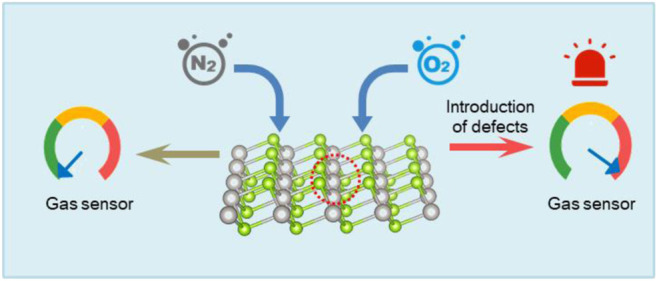


The generally used O_2_ and N_2_ gas sensors can be classified as electrical sensors and optical sensors according to the sensing principles: First, each kind of sensor requires efficient adsorption of the targeting gas molecules on the sensing material. For an electrical sensor, prominent charge transfer between the gas molecules and sensing material or charge trapping upon molecule adsorption converts the adsorption of gas molecules into electrical signal for detection. For an optical sensor, the adsorption of gas molecules notably alters the optical absorption spectrum of the sensing material. Traditionally, gas-sensing materials are metal–oxide–semiconductors, such as TiO_2_, SnO_2_, and ZnO (Kumar et al., 2014; Ibrahim et al., 2016; Xia et al., 2016). Recently, because of the theoretically infinite volume-to-surface ratio, which can provide enough active gas adsorption sites, intense studies on the gas-sensing properties of two-dimensional monolayer materials are reported (Yue et al., 2013; Bui et al., 2015; Ma et al., 2016a; Ma et al., 2016b; Sajjad et al., 2017; Klement et al., 2018; Ma X. et al., 2018; Ma D. et al., 2018; Jin et al., 2019; Ma D. et al., 2019; Ma et al., 2021). In particular, the intrinsic excellent sensing properties of Pt element render the monolayer PtSe_2_ as one of the mostly examined 2D gas-sensing material (Zhao et al., 2020). For example, Muhammad Sajjad et. al. studied the gas sensitivity of monolayer PtSe_2_ to the toxic NO_2_, NO, NH_3_, and CO gases (Sajjad et al., 2017); Dachang Chen et. al. studied the potential of PtSe_2_ as a gas sensor to detect SF_6_ decompositions (Chen et al., 2018).

On the other hand, the intrinsic defects, which can significantly affect the chemical, electrical, optical, and magnetic properties of PtSe_2_, are also extensively investigated. For example, Junfeng Gao et. al. studied the atomic structures and thermodynamic stability of vacancy defects. The study of Husong Zheng et. al. shows that the intrinsic Pt vacancy, Se vacancy, and Se@Pt anti-site defects can widely exist in ultrathin layered PtSe_2_ (Zheng et al., 2019); Ahmet Avsar et. al. found that Pt vacancy is responsible for the layer-dependent magnetism of PtSe_2_ (Avsar et al., 2019). In 2020, Jun Ge et. al. also reported the existence of magnetic moments induced by Pt vacancy defects in PtSe_2_ flakes (Ge et al., 2020).

Considering the ubiquity and easy introduction of intrinsic defects in PtSe_2_, in this work, we explore the N_2_ and O_2_ gas-sensing properties of both pure PtSe_2_ and PtSe_2_ with intrinsic defects, including Pt and Se vacancy defects (hereafter denoted as Pt-v and Se-v), Pt@Se and Se@Pt anti-site defects, and Pt and Se interstitial defects (hereafter denoted as Pt-inter and Se-inter), by first-principles calculations. It is found that PtSe_2_ with the Pt@Se anti-site defect has strong N_2_ adsorption capacity and exhibits significant change in the electrical properties upon N_2_ adsorption. Similar results are also observed for PtSe_2_ with Pt-v, Se-v, and Pt@Se defects when examining O_2_ adsorption. In addition, notable changes in the optical absorption spectra of the PtSe_2_ with Pt@Se defect are induced upon N_2_ adsorption, which also occurs for PtSe_2_ with Pt-v, Se-v, and Pt@Se upon O_2_ adsorption. These results demonstrate that PtSe_2_ with the corresponding defects can sensitively detect N_2_ and O_2_ molecules.

## Computational Methods

The first-principles calculations are conducted using the Vienna *Ab initio* Simulation Package (VASP) (Kresse and Furthmüller, 1996). A cutoff energy of 400 eV is used for plane wave expansion, and the accuracy for self-consistent iteration is set to 10^–5 ^eV. 4 × 4 supercells of pure PtSe_2_ are used for modeling the defected and molecules adsorbed on PtSe_2_, and the Brillouin zones for them are sampled with 3 × 3 × 1 gamma-centered k-points (Monkhorst and Pack, 1976). A vacuum layer larger than 30 Å is used for separating the atoms from their periodic images. For geometric optimization, the generalized gradient approximation (GGA) functional of Perdew–Burke–Ernzerhof is used (Perdew et al., 1996), and the atomic structures are fully relaxed until the residual forces on each atom are smaller than 0.02 eV/Å. To describe the interaction between the molecule and surface, DFT-D2 correction is used in the calculation. The more accurate HSE06 functional is used for calculating the electronic structures of pure and defected PtSe_2_ (Heyd et al., 2003). In order to quantify the electron charge redistribution between the adsorbed gas molecule and PtSe_2_, the Bader charge is analyzed based on the method of Henkelman (Henkelman et al., 2006; Sanville et al., 2007).

## Results and Discussion

### Adsorption Structures of Gas Molecules on Pure and Defected PtSe_2_

**FIGURE 1:**
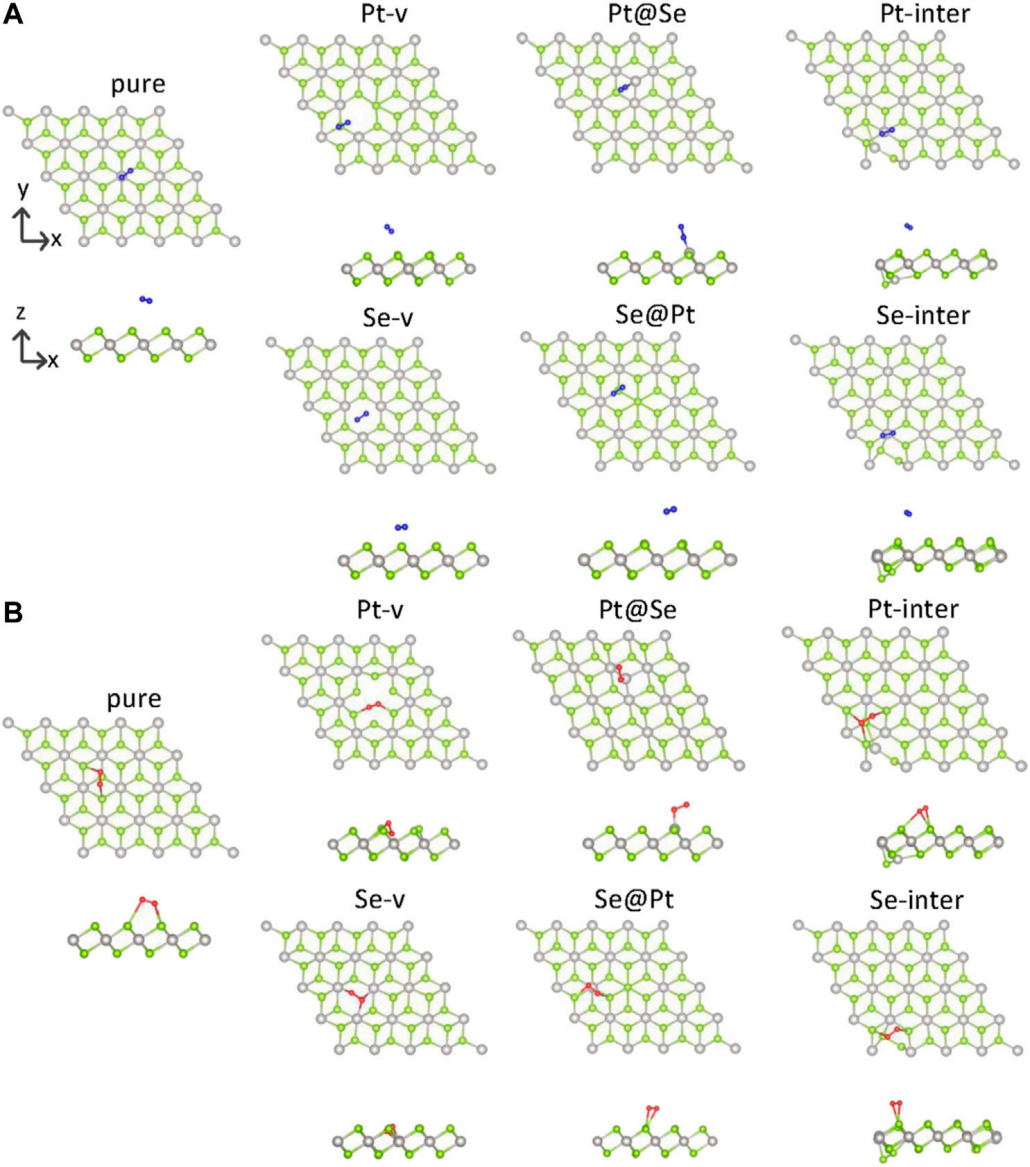
Top and side views of the adsorption structures of N_2_ **(A)** and O_2_ **(B)** on pure and defected PtSe_2_. The green and gray balls indicate Se and Pt atoms, respectively.

Adsorption of gas molecules on PtSe_2_ is an important parameter determining its gas-sensing properties. To establish the most stable adsorption structures of gas molecules on the monolayer, we first set many different configurations of gas molecules on the basal plane of PtSe_2_, which are then geometrically optimized. In this article, the Pt-v (Se-v) defect is formed by removing one Pt (Se) atom from a 4 × 4 supercell of pure PtSe_2_; the Pt@Se (Se@Pt) defect is formed by substituting Pt (Se) for Se (Pt), and the Pt-inter and Se-inter defects are formed by inserting Pt and Se atoms into the pure PtSe_2_. The structures with the largest adsorption energies are regarded as the most possible ones. The adsorption energy is defined as follows:

$$E_{\text{a}}=E_{\text{molecule}}+E_{\text{monolayer}}-E_{\text{total}},$$

where $E_{\text{molecule}}$ is the energy of an isolated gas molecule, $E_{\text{monolayer}}$ is the energy of pure and defected PtSe_2_, and $E_{\text{total}}$ is that of the molecule adsorbed system. The obtained most possible structures of nitrogen and oxygen adsorbed on pure and defected PtSe_2_ are shown in Figure 1. Figure 1A shows the adsorption structures of N_2_ on pure PtSe_2_ and PtSe_2_ with six kinds of intrinsic defects. Figure 1 shows the adsorption structures of O_2_ on pure PtSe_2_ and PtSe_2_ with the six kinds of intrinsic defects. In Table 1, we list the detailed adsorption energy, related bond lengths, and Bader charges on molecules for the adsorption structures shown in Figure 1. It is necessary to point out that the reason why the Pt-v defects in Figures 1A,B are different is that the atomic structures around the Pt-v defect change significantly upon adsorption of N_2_, whereas the changes in atomic structures are minor upon adsorption of O_2_.

**TABLE 1:** Adsorption distance *L* (in Å), the absorption energy *E*a (in eV), the change in the molecular bond length *Δ* (in Å) upon adsorption, and the values of Bader charges on molecules for the various adsorption structures.

	N_2_				O_2_			
	*L*(Å)	*E*_a_ (eV)	Δ(Å)	Bader(*e*)	L(Å)	*E*_a_ (eV)	Δ(Å)	Bader(*e*)
Pure	3.47	0.08	0.0004	0.02	2.85	0.51	0.0206	0.20
Pt-v	3.68	1.83	0.0002	0.02	1.86	2.36	0.2458	0.89
Se-v	3.58	0.19	0.0003	0.04	2.05	3.20	0.1693	0.80
Pt@Se	1.98	0.61	0.0142	0.14	1.92	1.98	0.0784	0.44
Se@Pt	3.55	0.08	0.0005	0.02	2.60	0.61	0.0297	0.28
Pt-inter	3.52	0.11	0.0005	0.02	2.73	0.59	0.0329	0.29
Se-inter	3.57	0.10	0.0005	0.02	2.91	0.52	0.0195	0.20

To describe the bonding length of the molecules on the PtSe_2_ surface, we define the adsorption distance *L* as the closest distance between the atoms of gas molecules and the surface atoms. As shown in Figure 1; Table 1, it is observed that the N_2_ molecule bonds with the surface Se atom of pure PtSe_2_ and the adsorption distance and absorption energy is, respectively, 3.47 Å and 0.08 eV, which is similar to the vdW interaction length between Se and N atoms. This indicates that N_2_ adsorbs on the surface by very weak vdW force. For the PtSe_2_ with Pt-v defect, N_2_ still bonds with the surface Se atom and the absorption energy becomes about 1.83 eV, which is much larger than that on pure PtSe_2_. This is because the atomic structures around the Pt-v defect change significantly upon adsorption of N_2_, which releases a significant amount of energy as will be discussed in later section. However, the adsorption distance is as long as 3.68 Å. For PtSe_2_ with Pt@Se anti-site defect, N_2_ adsorbs on the surface by forming N–Pt bond, and the bond length and absorption energy are, respectively, 1.98 Å and 0.61 eV, which is within the sum of atomic radii of N and Pt (2.33 Å), indicating chemical interaction between them. For PtSe_2_ with Se-v, Se@Pt, Pt-inter, and Se-inter defects, the N_2_ molecule still bonds with the surface Se atom and the adsorption energies are only slightly larger than those on pure PtSe_2_, and the adsorption distances are between 3.5 and 3.6 Å. Therefore, the interaction between these defected structures and N_2_ molecule is of the vdW nature.

As shown in Figure 1B; Table 1, O_2_ bonds with the surface Se atoms of pure PtSe_2_, and the corresponding adsorption distance and adsorption energy are, respectively, 2.85 Å and 0.51 eV, which is larger than the lengths of any chemical bonds between O and Se. This indicates that O_2_ adsorbs on the surface mainly by vdW force. For PtSe_2_ with Pt-v defect, the adsorption distance becomes 1.86 Å, which is similar to the sum of covalent radii of O and Se (1.89 Å), and the absorption energy is as large as 2.36 eV. This indicates that O_2_ is chemically bonded to the surface. For PtSe_2_ with Se-v and Pt@Se defects, the O_2_ molecule is bonded to the surface by forming one or more Pt–O chemical bonds, and the adsorption energies are, respectively, 3.20 and 1.98 eV, and the bond lengths are, respectively, 2.05 and 1.92 Å, which is smaller than the sum of atomic radii of O and Pt (2.25 Å). Notably, as shown in Figure 1B, the O_2_ molecule is deeply embedded in the vacancy sites of PtSe_2_ with Pt-v and Se-v defects, indicating strong adsorption of O_2_ on the surfaces. For PtSe_2_ with Se@Pt, Pt-inter, and Se-inter defects, O_2_ still bonds with the surface Se atom, and the adsorption energies are only slightly larger than those on the pure surface and the adsorption distances are between 2.60 and 2.91 Å. Therefore, the interaction between these defect structures and O_2_ molecules is of vdW nature.

From the above results, it is noted that O_2_ and N_2_ molecules only weakly adsorb on the pure PtSe_2_, while all the intrinsic defects enhance more or less the interaction between the gas molecules and PtSe_2_. In particular, the Pt@Se anti-site defect transforms the initially weak vdW interaction into a strong chemical interaction between the molecules and PtSe_2_, and the Pt-v and Se-v defects also result in strong chemical interactions between O_2_ and PtSe_2_. In addition, O_2_ adsorbs more strongly than N_2_ on both the pure and defected PtSe_2_. These results are also supported by the changes in the molecular bond lengths and the Bader charges on the molecules, as listed in Table 1.

### Charge Transfer and Electronic Structures of Gas Molecules on Pure and Defected PtSe_2_

**FIGURE 2:**
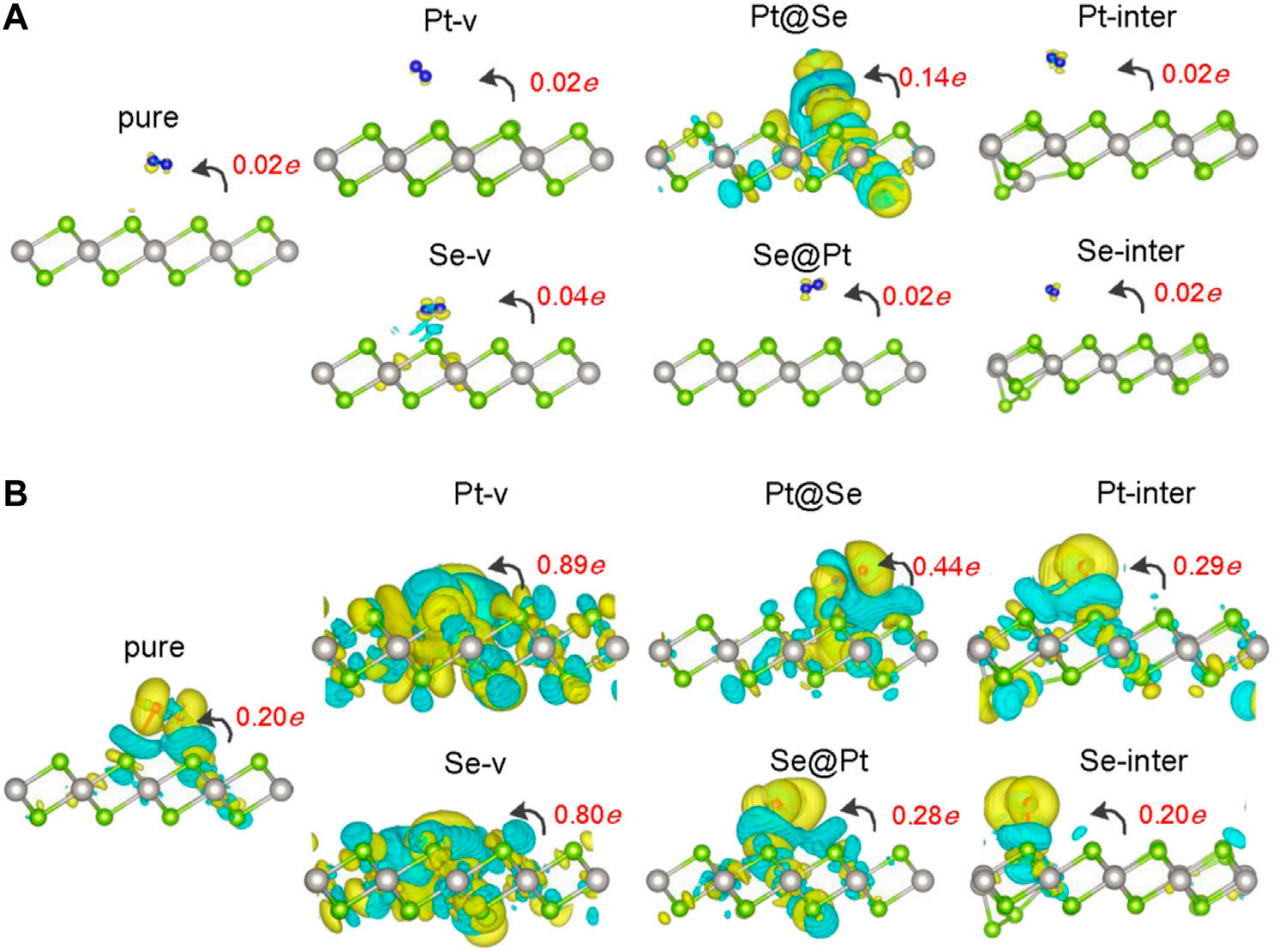
Charge density difference (CDD) for N_2_ **(A)** and O_2_ **(B)** adsorbed pure and defected PtSe_2_. The yellow (cyan) region represents charge accumulation (depletion), and the values of isosurfaces for all the structures are set to 0.0003 *e*/Å^−3^. The red numbers indicate the values of Bader charges on the molecules, and the black curved arrows indicate the orientation of charge transfer.

Prominent charge transfer between the gas molecules and PtSe_2_ upon molecule adsorption is a fundamental prerequisite for transforming the existence of a gas molecule into electrical signal during gas-sensing application. To investigate the charge transfer between them, the Bader charge on molecules and charge density difference (CDD) for the molecule adsorbed on pure and defected PtSe_2_ are calculated and shown in Figure 2. The CCD is calculated according to the following equation:

$$\Delta_{\rho }=\rho_{\text{total }}-\rho_{\text{monolayer }}-\rho_{\text{molecule }},$$

where $\rho_{\text{total }}$, $\rho_{\text{monolayer }}$, and $\rho_{\text{molecule }}$ are the charge densities of the molecule-adsorbed system, pure or defected PtSe_2_ without molecule adsorption, and the isolated gas molecule, respectively. From Figure 2, it is noted that there is always charge transfer from PtSe_2_ to N_2_ and O_2_ upon molecule adsorption, except that the charge transfer between N_2_ and pure PtSe_2_ is very weak, with a Bader charge of 0.02 *e*. For PtSe_2_ with Pt-v, Se-v, Se@Pt, Pt-inter, and Se-inter defects, the charge transfer between N_2_ and them is almost the same as that between N_2_ and pure PtSe_2_, whereas the Pt@Se anti-site defect significantly promotes charge transfer from PtSe_2_ to N_2_, with a Bader charge of 0.14 *e*. Moreover, the results of CDD show that significant charge redistribution around the Pt@Se anti-site defect also occurs upon adsorption of N_2_, suggesting additional charge trapping effect of the defect. For O_2_ on pure PtSe_2_ (Figure 2B), the amount of charge transfer is relatively large (about 0.20 *e*), and the introduction of Pt-v, Se-v, and Pt@Se defects further increases the amount of charge transfer, with Bader charges being up to 0.89 *e*, 0.80 *e*, and 0.44 *e*, respectively. For PtSe_2_ with Se@Pt, Pt-inter, and Se-inter defects, the charge transfer is only slightly larger than that on pure PtSe_2_, with Bader charges between 0.20 *e* and 0.29 *e*. Similarly, the results of CDD in Figure 2 show that significant charge redistribution around the various intrinsic defects occurs upon adsorption of O_2_, suggesting additional charge trapping effects of the defects.

**FIGURE 3:**
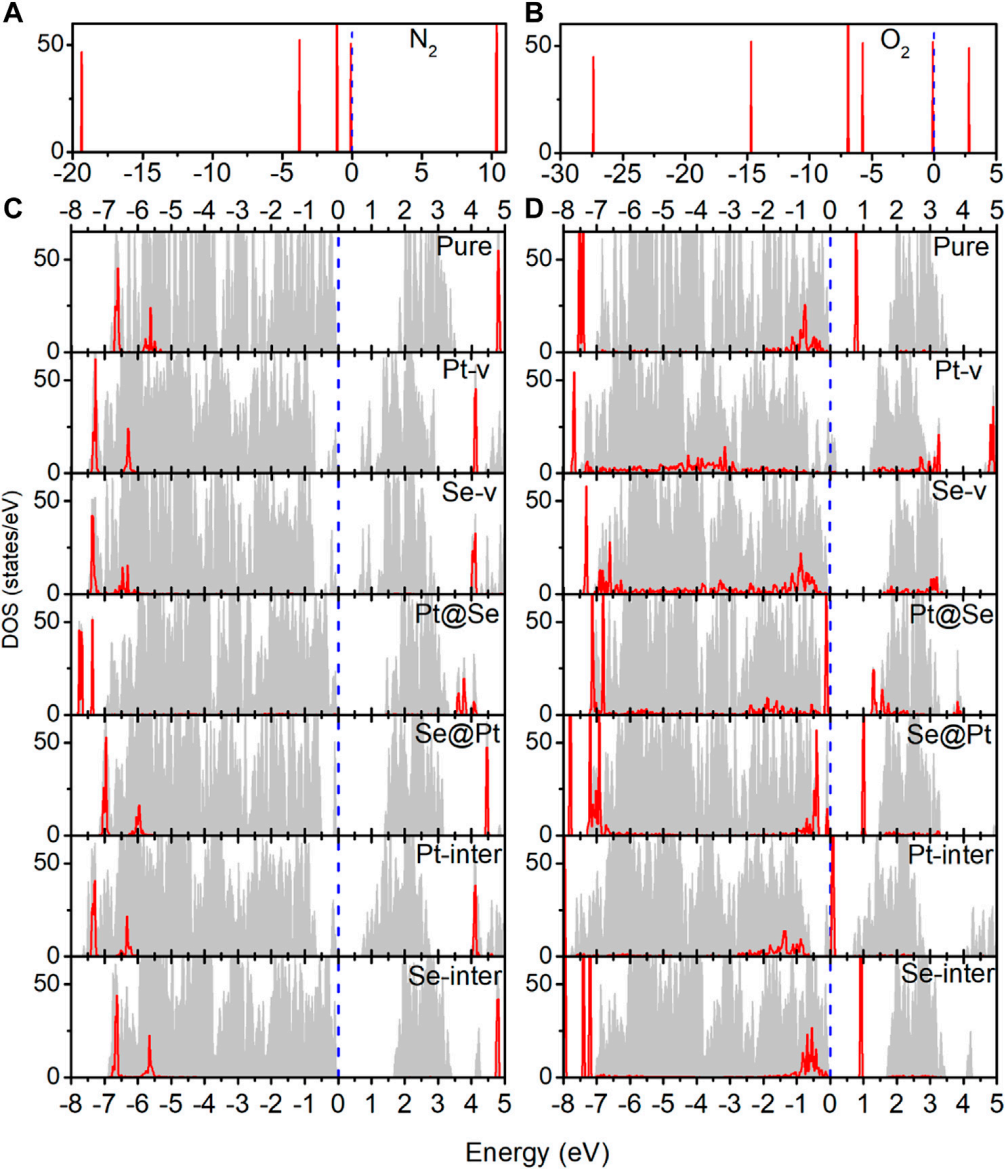
**(A)** Density of states (DOS) of the isolated N_2_ molecule. **(B)** DOS of the isolated O_2_ molecule. **(C)** DOS of pure and defected PtSe_2_ with the adsorption of N_2_. **(D)** DOS of pure and defected PtSe_2_ with the adsorption of O_2_. The gray areas indicate total DOS of the corresponding structures, and the red lines indicate projected DOS of the absorbed molecule. The values of DOS for adsorbed N_2_ and O_2_ on PtSe_2_ are set to 1.5 and 4 times for clear comparison. The Fermi levels (blue-dashed lines) for all the structures are set to 0 eV.

**FIGURE 4:**
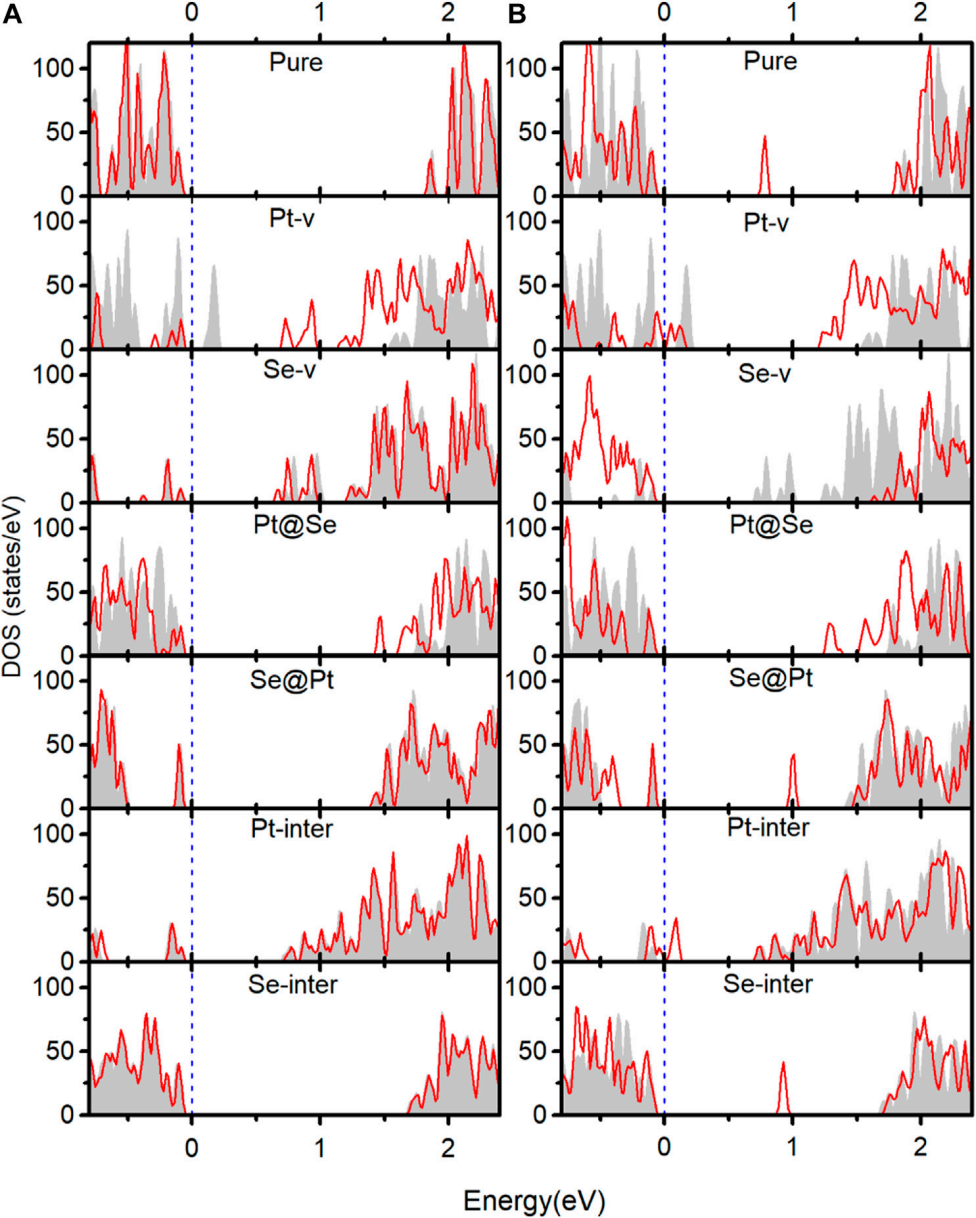
Red lines indicate the DOS near the band edges of pure and defected PtSe_2_ with the adsorption of N_2_ **(A)** and O_2_ **(B)**. The gray areas indicate the DOS near band edges of pure and defected PtSe_2_.

In order to understand the adsorption structures, charge transfer between molecules and PtSe_2_, and the charge trapping effects of the intrinsic defects shown above, we investigate the density of states (DOS) for pure and defected PtSe_2_ with the adsorption of gas molecules, the DOS of isolated N_2_ and O_2_ molecules, and the DOS of pure and defected PtSe_2_. As shown in Figure 3, the DOS of isolated N_2_ molecules shows that the 2*π* bonding orbital just lies under the Fermi level and has a lower height than 5σ orbital near −1 eV. The DOS of N_2_ basically still retains the characteristics of isolated N_2_ after adsorption on pure PtSe_2_ and PtSe_2_ with Pt-v, Se-v, Se@Pt, Pt-inter, and Se-inter defects, which is consistent with the weak interaction between N_2_ and pure and the defected PtSe_2_, whereas the DOS of N_2_ changes significantly upon the adsorption on PtSe_2_ with the Pt@Se anti-site defect. For example, the antibonding orbital of N_2_ near 4 eV splits into three peaks with lower height, and the relative values of the two bonding orbitals near −7.5 eV reverse, and they are in resonance with electronic states from PtSe_2_, indicating strong chemical interaction between N_2_ and PtSe_2_. In addition, because N_2_ bonds with the PtSe_2_ surface by forming Pt–N chemical bonds, the large electronegativity of N results in notable electron gain from Pt, which leads to the large value of Bader charge on N_2_. On the other hand, as shown in Figure 4, the electronic states near the valence band maximum (VBM) and conduction band minimum (CBM) are almost unchanged upon adsorption of N_2_ on pure PtSe_2_ and PtSe_2_ with Se-v, Se@Pt, Pt-inter, and Se-inter defects, whereas small gap states near both CBM and VBM appear upon adsorption of N_2_ on the PtSe_2_ with Pt@Se defect, which may additionally trap electrons and holes. These are consistent with the results of CDD shown above. For the PtSe_2_ with Pt-v defect, the atomic structures around the defect significantly change upon adsorption of N_2_, which introduces many gap states, as will be discussed in the following section.

As shown in Figure 3B, the DOS of isolated O_2_ shows five peaks lower than the Fermi level, and the 2π* antibonding orbital is the highest occupied orbital. The DOS of O_2_ hybridizes notably and to different extent with the DOS of PtSe_2_ upon adsorption on pure PtSe_2_ and defected PtSe_2_, and the hybridization is especially significant on PtSe_2_ with Pt-v, Se-v, and Pt@Se defects. These are consistent with the strong and different interactions between O_2_ and pure and defected PtSe_2_. Moreover, because O_2_ bonds with the PtSe_2_ surface by forming Pt (Se)–O vdW or chemical bonds, the large electronegativity of O results in notable electron gain, which leads to the large values of Bader charges on O_2_. On the other hand, as shown in Figure 4B, new electronic states near the VBM and CBM are introduced upon adsorption of O_2_ on pure PtSe_2_ and all the defected PtSe_2_, which may additionally trap electrons and holes. Therefore, significant redistribution of charge density around the defects occurs, as shown in the CDD of Figure 2B.

From the results above, it is noted that for N_2_, it is mainly the Pt@Se anti-site defect that can notably enhance the charge transfer between the gas molecules and PtSe_2_ and charge trapping states, while the other defects show negligible effects. For O_2_, all the defects, except Se-inter, enhance the charge transfer between the molecule and PtSe_2_, and the effects of Pt-v, Se-v, and Pt@Se defects are the most significant. In addition, the intrinsic defects introduce new electron and/or hole trapping states near the VBM and/or CBM. The charge transfer and charge trapping effects can result in significant electric signal when the defected PtSe_2_ is used as electrical sensors.

## Optical Absorption Properties of Pure and Defected PtSe2 With Molecule Adsorption

**FIGURE 5:**
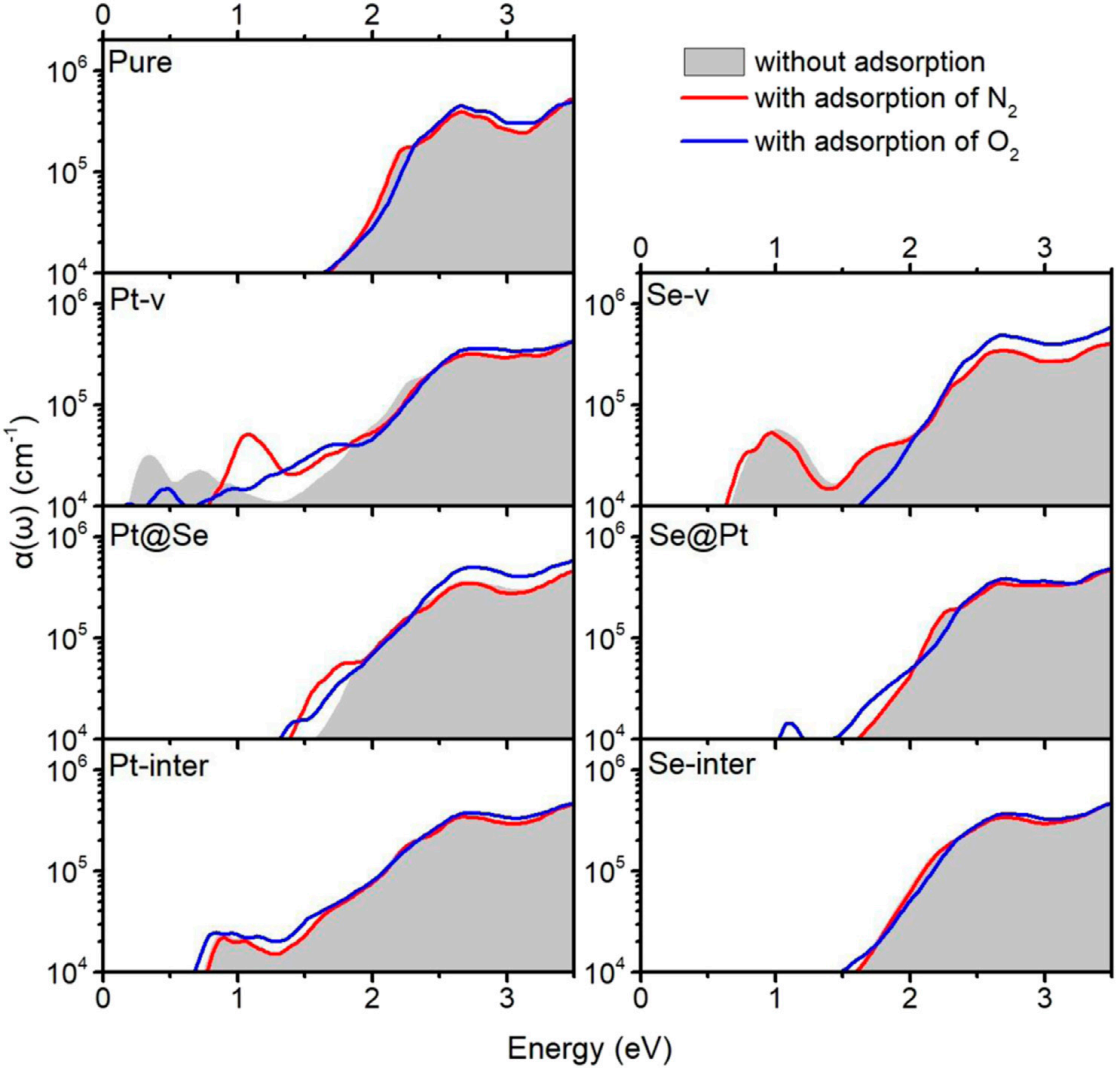
Optical absorption coefficients α(ω) of pure PtSe_2_ and defected PtSe_2_ with adsorption of N_2_ (red line) and O_2_ (blue line). The gray areas indicate the optical absorption coefficients of pure PtSe_2_ and defected PtSe_2_.

The intrinsic defects and molecule adsorption not only affect the electronic structure and electrical properties of PtSe_2_ but may also affect its optical absorption properties. In order to study how they affect the optical properties of PtSe_2_, we calculated the optical absorption coefficients of pure and defected PtSe_2_ absorbed with N_2_ and O_2_. The specific calculation procedure is the same as one of our previous works (Ma X. et al., 2018; Ma X. et al., 2019; Yong et al., 2020; Jian et al., 2021). Figure 5 shows the optical absorption coefficients for polarization of E field along the in-plane *x* direction of pure and defected PtSe_2_ with and without the adsorption of N_2_ and O_2_. Note that the results for the polarization of E field along the in-plane *y* direction are almost the same as those along the in-plane *x* direction. As shown in Figure 5, for adsorption of N_2_, the optical absorption coefficients of pure PtSe_2_ and PtSe_2_ with Se-v, Se@Pt, Pt-inter, and Se-inter defects are basically the same as those of the structures without N_2_ adsorption. For the PtSe_2_ with Pt-v defect, there are two absorption peaks around 0.33 and 0.72 eV, whereas the adsorption of N_2_ eliminates these two absorption peaks and introduces a new and prominent absorption peak around 1.07 eV. For the PtSe_2_ with Pt@Se defect, the optical absorption edges are slightly extended to lower energy.

On the other hand, for adsorption of O_2_, the optical absorption coefficients of pure PtSe_2_ and PtSe_2_ with Pt-inter and Se-inter defects are roughly the same as those of the corresponding structures without adsorption. For the PtSe_2_ with Pt-v defect, the initial two absorption peaks around 0.33 and 0.72 eV are weakened and the absorption valley at 1.3 eV is filled upon the adsorption of O_2_. For the PtSe_2_ with Se-v defect, the notable absorption peak around 1.0 eV is quenched, and the optical absorption edge blue shifts significantly upon O_2_ adsorption. For PtSe_2_ with Pt@Se and Se@Pt defects, the optical absorption edges, mainly red shift slightly upon O_2_ adsorption. The notable changes in the absorption coefficients of defected PtSe_2_ upon N_2_ and O_2_ adsorption further verify the significant interactions between them and suggest that the characteristic changes in the optical spectra may be utilized for making high-performance and sensitive optical N_2_ and O_2_ gas detectors.

## Special Results of Pt-v Defect Introduced by N_2_ Adsorption

**FIGURE 6:**
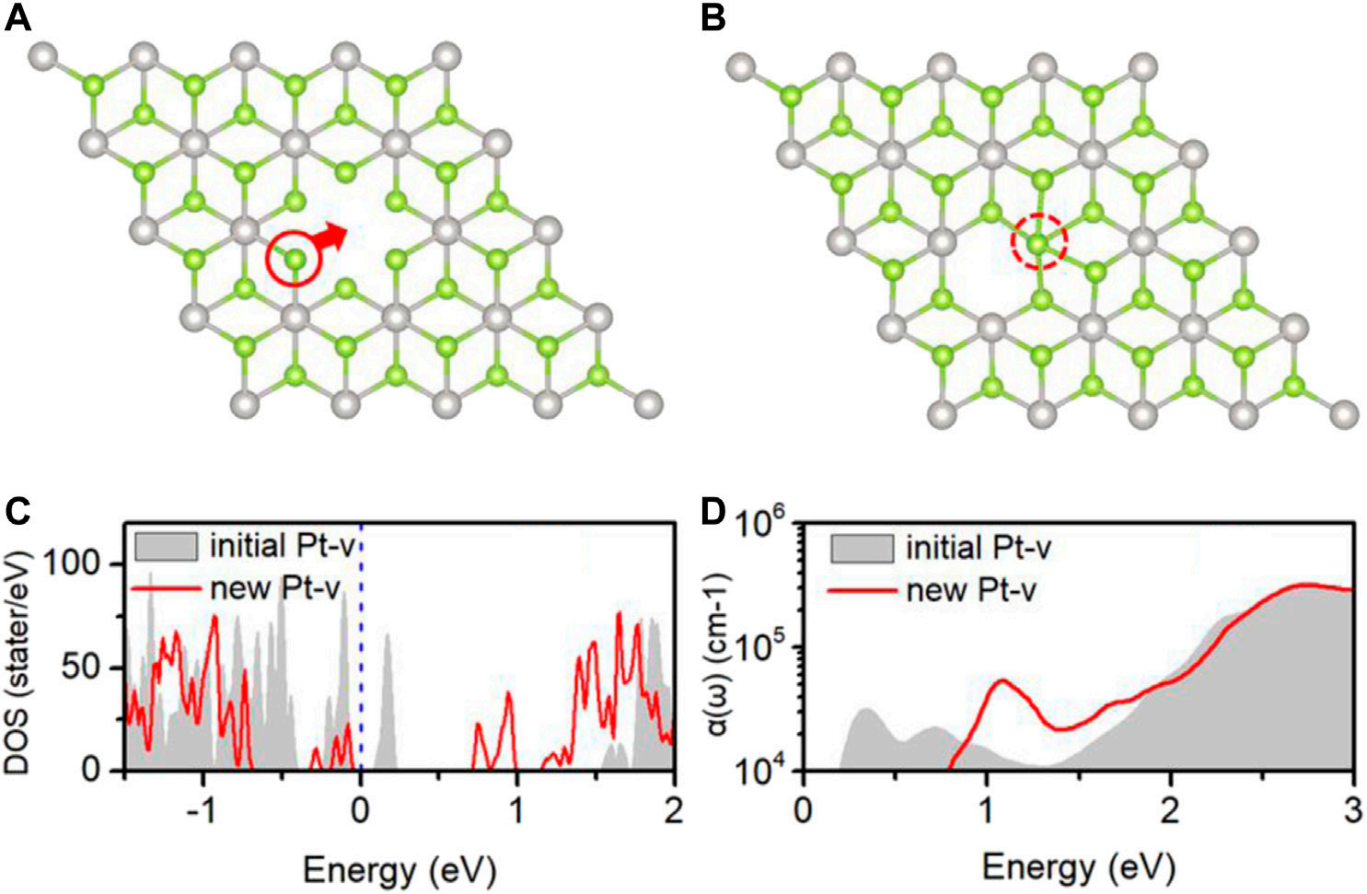
**(A)** Local atomic structures of the initial **(A)** and new **(B)** Pt-v defects; the DOS **(C)** and optical absorption coefficient **(D)** of PtSe_2_ with the initial Pt-v defect (gray areas) and new Pt-v defect (red lines). The Fermi levels (blue dashed line) are set to 0 eV.

Figure 6A shows the initial structures of the PtSe_2_ with Pt-v defect, which is formed by removing one of the Pt atoms in the supercell model and first-principles optimization. Upon N_2_ adsorption, the surrounding atomic structures of the Pt-v vacancy site change significantly, and these changes are retained when removing the adsorbed N_2_ molecule. As shown in Figure 6B, the upper Se atom closest to the defect site moves to the original position of Pt vacancy and bonds with the surrounding Se atoms. To characterize the differences in the properties of the two structures, the electronic structure and optical properties of them are calculated and shown in Figures 6C,D. As can be seen, the introduced gap states and the DOS near the valence band edge are very different for the two structures. The initial Pt-v structure introduces both occupied and unoccupied gap states near the valence band edge, whereas the new Pt-v structure introduces occupied gap states near the valence band edge and unoccupied gap states near the conduction band edge, thus exhibiting very different electrical properties. Because of this, the optical absorptions of them also show different characteristics. As shown in Figure 6D, the gray area shows that there are two absorption peaks around 0.33 and 0.72 eV, resulting from the gap states near the Fermi level, and there is an absorption valley at 1.3 eV. For the new Pt-v structure, there is mainly a characteristic absorption peak at 1.1 eV, resulting from the transition between gap states. The different optical absorption properties of the two structures may be used to differentiate the specific atomic structures of the Pt-v defect. The recent studies have shown that in few-layer PtSe_2_ flakes, the Pt vacancy defect on the surface and inside can produce localized magnetic moments. The versatile properties of Pt-v and easy tunability of Pt-v with the adsorption of N_2_ revealed here may be used to understand and tune the magnetic properties of PtSe_2_.

## Conclusion

In conclusion, we have studied the N_2_ and O_2_ gas-sensing properties of monolayer PtSe_2_ by characterizing the geometric structures, charge transfer, electronic structures, and the optical absorption of pure and defected PtSe_2_ with and without the adsorption of N_2_ and O_2_ molecules. It is found that both N_2_ and O_2_ adsorb weakly on pure PtSe_2_, whereas the Pt@Se anti-site defect significantly improves the N_2_ adsorption capacity of PtSe_2_ by converting the initial weak vdW interaction on pure PtSe_2_ into strong chemical interaction. Moreover, the defect not only promotes charge transfer from PtSe_2_ to N_2_ but also introduces charge trapping states around the defect, which leads to a significant change in the electrical properties of the structure. Similar results are also observed for the Pt-v, Se-v, and Pt@Se defects when examining O_2_ adsorption. In addition, a notable change in the optical absorption spectra of the PtSe_2_ with Pt@Se defect is induced upon N_2_ adsorption, and this also occurs for PtSe_2_ with Pt-v, Se-v, and Pt@Se upon O_2_ adsorption. Therefore, PtSe_2_ with the corresponding defects are promising materials for preparing sensitive electrical and optical sensors for detecting N_2_ and O_2_ molecules. Our work demonstrates the important role of intrinsic defects in improving and extending the sensing performance of PtSe_2_, which may be generalized to other materials.

Surprisingly, it is also found that significant changes in the atomic structures around Pt vacancy defect are induced upon adsorption of N_2_, which results in very different electronic and optical properties. The versatile properties of Pt vacancy and easy tunability with N_2_ molecules revealed here may have potential application for understanding and tuning the recently reported magnetic properties of PtSe_2_.

## Data Availability

The original contributions presented in the study are included in the article/Supplementary Material, and further inquiries can be directed to the corresponding author.
